# A Case of Neonatal Alloimmune Thrombocytopenia Following Maternal Pemphigoid Gestationis

**DOI:** 10.7759/cureus.44946

**Published:** 2023-09-09

**Authors:** Danielle A Chism, Kaila R Fives, Bailey Beetz, Marc Berger

**Affiliations:** 1 Medicine, Lake Erie College of Osteopathic Medicine, Bradenton, USA; 2 Pediatrics, Baptist Health, Jacksonville, USA

**Keywords:** neonate, thrombocytopenia, immunology, pemphigus, neonatal alloimmune thrombocytopenia

## Abstract

Neonatal alloimmune thrombocytopenia (NAIT) is a complex condition, stemming from the transplacental passage of alloantibodies from a pregnant mother directed against fetal platelet antigens. This case report discusses a rare instance of severe NAIT initially presenting as inadequate weight gain. After a clinical workup yielded negative findings for an infection and the resolution of the patient's thrombocytopenia following the administration of platelet products and intravenous immunoglobulin (IVIG), hematology deduced that this patient’s NAIT was secondary to maternal history of gestational pemphigus. We describe the pathophysiology and current understanding of NAIT, pemphigoid gestationis (PG), as well as an analysis of their association. This intersection of NAIT and maternal PG underscores the importance of considering potential interactions between maternal autoimmune conditions overall and their impact on fetal health.

## Introduction

Neonatal alloimmune thrombocytopenia (NAIT) arises from the transplacental transfer of alloantibodies produced by a pregnant mother that are directed against fetal platelet antigens. This mechanism is similar to the process seen in ABO blood incompatibility, where preformed maternal antibodies attack fetal red blood cell antigens leading to hemolytic disease in the newborn. The incidence of NAIT is one in every 1000 live births in the United States, and it is the most common cause of thrombocytopenia with platelet counts reaching below 30k (normal range: 150-450k) [1]. A low platelet count can cause serious bleeding complications, most notably intraventricular hemorrhage (IVH). The most common platelet antigen involved is human platelet antigen (HPA) 1a, but any platelet antigen can be involved. This antigen is present in about 90% of the population, but among the HPA-1a-negative women, the likelihood of developing immunization is mainly associated with human leukocyte antigen (HLA)-DR3 [2]. Effective management strategies include platelet transfusion and intravenous immunoglobulin (IVIG) therapy [2,3].

Pemphigoid gestationis (PG) is an autoimmune skin disorder that manifests during pregnancy and is characterized by skin vesicles and bullae that typically spares the face and mucous membranes. While the timing of its onset varies, it most frequently presents with skin lesions and severe pruritis during the third trimester. The rash also worsens during labor or immediately postpartum in up to 75% of women [4]. The underlying pathophysiology of PG, like other pemphigoid diseases, involves the production of IgG autoantibodies targeting hemidesmosomal proteins of the basement membrane. The abnormal expression of placental HLA class II antigens exacerbates this process. Specifically, PG is known to be strongly associated with HLA-DR3 and HLA-DR4. The fetal risks in PG are prematurity, low birth weight, and temporary skin lesions. Thankfully, the condition is not associated with an increased risk of stillbirth or abortion [5].

We present an interesting case involving the development of NAIT in an infant who originally presented due to inadequate weight gain. Given the mother's history of PG, we believe that the antibodies produced from the mother's third-trimester gestational pemphigus crossed the placenta and targeted the neonate’s platelets. We also explore the pathophysiology of both conditions and investigate the possibility of immunological interaction between the two conditions.

## Case presentation

The patient was an eight-day-old male who presented to the hospital due to a failure to thrive (FTT). He was a 35-week-old premature infant delivered via normal vaginal spontaneous delivery. He had weighed 2630 g at birth and had been discharged from the newborn nursery two days later at 2400g, still within the range of normal weight loss in a neonate (8.6%; normal range: 7-10%). The patient was brought for his neonatal pediatrician appointment at eight days of life (DoL) weighing only 2430g, still 7.6% lower than his birth weight. The mother expressed concern because the patient was not adequately gaining weight despite being formula-fed. Hence, he was admitted to the hospital with a primary diagnosis of FTT in the neonate. Maternal history revealed that the mother had been diagnosed with PG during the third trimester of her pregnancy.

On the first night of the hospital stay, the patient had an episode of desaturation with periodic breathing as well as a fever. He was quickly worked up for neonatal sepsis and was given ampicillin, gentamicin, and acyclovir empirically to cover for the most common bacterial and viral causes of neonatal meningitis. A complete blood count (CBC) revealed thrombocytopenia with a value of 18 K/mcL (Table 1). The patient was transferred to the PICU for closer observation and arrived at the medical unit in stable condition.

**Table 1:** Labs upon admission

Labs on 07/08/2021 @ 10:05	
Red blood cell count	3.67 mil/mcL (low)
Hematocrit	38.6% (low)
Platelet count	18 K/mcL (critical)
Basophils	4% (high)
Basophils absolute	0.26 k/mcL (high)
Myelocytes %	2% (high)
Platelet sufficiency	Decreased
Large platelets	Present
Ovalocytes	Few

The results of his cerebral spinal fluid (CSF), blood, and urine cultures were negative. He also tested negative for cytomegalovirus (CMV) and other “TORCH” infections, including toxoplasmosis, syphilis, rubella, herpes, and HIV, which prompted the cessation of the antibiotics and acyclovir at the time of the results. Initial labs also revealed transaminitis, prompting an ultrasound of the liver and biliary structures to rule out pathologies associated with those structures. The results showed nonspecific gallbladder thickening and a normal liver. Due to his continued low platelet count on repeat labs 3.5 hours after the initial set (Table 2), hematology was consulted and they advised that the patient be given three doses of IVIG as well as two units of platelet transfusions.

**Table 2:** Repeat labs

Labs on 07/08/2021 @ 13:34	
Platelet count	29 K/mcL (low)
Protein urine	30 mg/dL
Blood urine	Trace
Bacteria urine	Rare

Hematology concluded that since the mother had pemphigus associated with pregnancy, the placental crossing of antibodies from PG likely caused thrombocytopenia in the patient. An ultrasound of the head was also performed due to concerns for IVH secondary to critical thrombocytopenia and prematurity, and it was also negative. After undergoing the aforementioned treatments, the patient's platelet count at discharge was 176K/mcL; he was stable, and a plan was discussed to follow up with hematology in two weeks for repeat CBC and re-examination.

## Discussion

The diagnostic approach must be prompt when there is an initial suspicion of FTT in a neonate. Our patient received a full workup, including blood, urine, and CSF cultures, CBC, blood chemistry, TORCH study, and liver function tests. When the evidence of infection turned out to be consistently negative and further workup regarding the transaminitis did not raise any concerns, the differential focus shifted toward the patient's thrombocytopenia of 18,000/mcL. 

Critically low levels of platelets in a neonate can manifest as intracranial bleeds, neurological sequelae, organ failure, and even death. Thrombocytopenia can occur due to a variety of pathologies, but of particular interest with regard to this case is platelet destruction. In immune thrombocytopenia, this destruction is initiated by antibodies that coat the surface of the platelet to opsonize them, ultimately leading to their demise by macrophages [6]. An asymptomatic mother with a symptomatic neonate should raise suspicion of NAIT. NAIT is caused by maternal immunoglobulin G alloantibodies that cross the placenta and are directed against HPAs on fetal platelets [7]. While the HPA antigens frequently involve maternal alloantibodies against HPA, human leucocyte antigen (HLA) antibody binding to platelets is a rare cause of NAIT [8]. In the fetal blood, the alloantibodies attack the platelet surfaces, as mentioned above, and neonatal thrombocytopenia ensues [6,7]. NAIT is one of the major causes of both severe thrombocytopenia and intracranial fetal hemorrhage, and its treatment involves IVIG therapy and platelet replacement [7].

The mother of the patient had no significant medical history other than PG. In PG, the pathophysiology involves the presence of IgG autoantibodies against the basement membrane of the skin [5]. This antibody created an autoimmune reaction against BP180, which is not only expressed in the skin but can also react against the fetoplacental unit [5]. Due to the passive transfer of antibodies from mother to fetus, 10% of newborns of mothers with PG develop clinical symptoms, including urticaria or vesicular skin lesions, and have an increased risk of premature delivery and low birth weight [9]. Adverse pregnancy outcomes appear to be more closely associated with higher antibody titers in maternal serum and neonatal cord blood [9]. The current proposed pathways involve two theories. One theory suggests that the existence of paternal antigens from the second class of major histocompatibility complex on chorionic villi generates an immune response from the mother and results in antibodies against the amniotic basement membrane. This theory suggests that these antibodies can cross-react with various antigens from the mom as well as induce fetal disease. The second hypothesis suggests a genetic predisposition, as studies have shown a possible connection between PG and second-class HLA [9].

Research on PG has suggested that it is strongly associated with maternal HLA-DR3 and HLA-DR4 [5]. This strong association indicated the important role of MHC class II in the pathogenesis of the disease. The condition usually presents as an eruption of eczematous or erythema multiforme-like lesions, erythematous urticarial plaques, and papules that can progress to vesicles, tense blisters, and bullae in over 65% of cases [5]. Diagnosis of PG is based on clinical symptoms and signs, skin biopsy and direct immunofluorescence, and serum level of BP180 antibodies using enzyme-linked immunosorbent assay (ELISA) [5]. Current studies state that PG is a rare disease that does not pose a major health burden. A few studies have linked PG with premature birth, fetal adrenal suppression, and various minor symptoms that resolve spontaneously within a few weeks. However, there is not enough evidence to sustain a link between PG and the previously mentioned sequelae.

In a study by Colvin et al. (2023), NAIT cases were found to have significantly higher HLA antibody strength as measured by mean fluorescence index, and broader HLA antibody specificity at antigen epitope level, compared to matched controls [10]. This study showed that more research is needed to examine further whether the strong HLA antibodies identified in HPA-antibody-negative cases directly cause neonatal thrombocytopenia and whether prenatal treatment may be warranted in select cases to prevent occurrence [10]. HLA antibody-induced NAIT should be suspected in a thrombocytopenic newborn with or without signs of bleeding within the first three days of life, especially when the newborn does not present with any illness [8]. In order to provide individualized care and anticipate NAIT in future pregnancies, follow-up maternal HLA antibody levels should be tested.

In ill-appearing, premature infants, common early-onset (defined as less than 24 hours of life) causes of thrombocytopenia are sepsis, TORCH infections, birth asphyxia, disseminated intravascular coagulation (DIC), and necrotizing enterocolitis (NEC). For late-onset premature infants (defined as greater than 72 hours of life), common causes of thrombocytopenia are sepsis, thrombosis, DIC, and drug-induced. Rarer causes include chromosomal disorder, inborn errors of metabolism, and Fanconi anemia. According to the literature, thrombocytopenia in infants is mostly due to infection, sepsis, or thrombosis, but our patient did not indicate any signs that these etiologies were the cause of his thrombocytopenia [11]. In this case, the lack of culture infection led to a hematology consult, which deduced an alloimmunization mechanism resulting from maternal antibodies. An improvement in symptoms with IVIG and platelet transfusion led hematologists to diagnose our patient with NAIT. Given the absence of any signs of infection, the maternal PG likely affected the patient's hospital course. Although we do not have the maternal HLA antibody levels, this patient’s hospital course endorses a diagnosis of NAIT secondary to PG. More studies need to be conducted to gain deeper insights into the association between HLA antibody levels in PG and the development of NAIT.

NAIT is normally identified when clinical signs of bleeding are evident shortly after birth or a platelet count confirms isolated thrombocytopenia. The immediate treatment for very severe thrombocytopenia (<30 x 102/L) involves platelet transfusion, and intravenous immunoglobulin (IVIG) can be given to potentially prolong the survival of the incompatible platelets and lower the overall period of thrombocytopenia [12]. In moderately severe thrombocytopenia (30-50 x 102/L) without obvious signs of hemorrhage, NAIT can be managed with IVIG alone [12]. NAIT in infants born subsequently to a mother who previously gave birth to an infant with this condition tends to be more severe [12]. Therefore, later pregnancies should be managed with physicians familiar with and experienced in NAIT diagnosis and treatment. This case highlights the importance of antibody testing to gain awareness of and anticipate NAIT cases in subsequent pregnancies for mothers with infants affected by NAIT.

## Conclusions

Considering the immunological pathophysiology of PG, further studies are needed to better understand the mechanism of HLA antibodies from PG leading to NAIT. We discussed a case of NAIT diagnosed in an infant following maternal PG. Further maternal antibody testing would enable providers to anticipate the need for administering IVIG and platelet products after birth. A limitation of this study is the lack of antibody testing in this case, possibly due to a lack of resources. However, based on the clinical diagnosis and effective treatment, we can deduce that NAIT was correctly diagnosed in this case. Antibody testing and fetal ultrasound will be crucial in anticipating and preventing the serious consequences of NAIT, which are thrombocytopenia leading to bleeding and possible IVH. More studies must be conducted on the association between maternal pemphigus and NAIT, with a specific focus on the HLA antibodies involved in both conditions and how they may interact.
